# N-Terminal Ubiquitination of Amyloidogenic Proteins Triggers Removal of Their Oligomers by the Proteasome Holoenzyme

**DOI:** 10.1016/j.jmb.2019.08.021

**Published:** 2020-01-17

**Authors:** Yu Ye, David Klenerman, Daniel Finley

**Affiliations:** 1 Department of Cell Biology, Harvard Medical School, Boston, MA 02115, USA; 2 Department of Chemistry, University of Cambridge, Cambridge CB2 1EW, UK; 3 UK Dementia Research Institute at Imperial College London, London W12 0NN, UK; 4 UK Dementia Research Institute at the University of Cambridge, Cambridge CB2 0XY, UK

## Abstract

Aggregation of amyloidogenic proteins is an abnormal biological process implicated in neurodegenerative disorders. Whereas the aggregation process of amyloid-forming proteins has been studied extensively, the mechanism of aggregate removal is poorly understood. We recently demonstrated that proteasomes could fragment filamentous aggregates into smaller entities, restricting aggregate size [1]. Here, we show *in vitro* that UBE2W can modify the N-terminus of both α-synuclein and a tau tetra-repeat domain with a single ubiquitin. We demonstrate that an engineered N-terminal ubiquitin modification changes the aggregation process of both proteins, resulting in the formation of structurally distinct aggregates. Single-molecule approaches further reveal that the proteasome can target soluble oligomers assembled from ubiquitin-modified proteins independently of its peptidase activity, consistent with our recently reported fibril-fragmenting activity. Based on these results, we propose that proteasomes are able to target oligomers assembled from N-terminally ubiquitinated proteins. Our data suggest a possible disassembly mechanism by which N-terminal ubiquitination and the proteasome may together impede aggregate formation.

## Highlights

• Amyloid proteins α-synuclein and tau^K18^ can be ubiquitinated by UBE2W.
• N-terminal ubiquitin modification on amyloid proteins delays aggregation.
• Proteasomes can remove N-terminal ubiquitin-modified oligomers.
• Proteasomes remove oligomers primarily by enabling their dissociation.

## Graphical abstract

**Figure:**
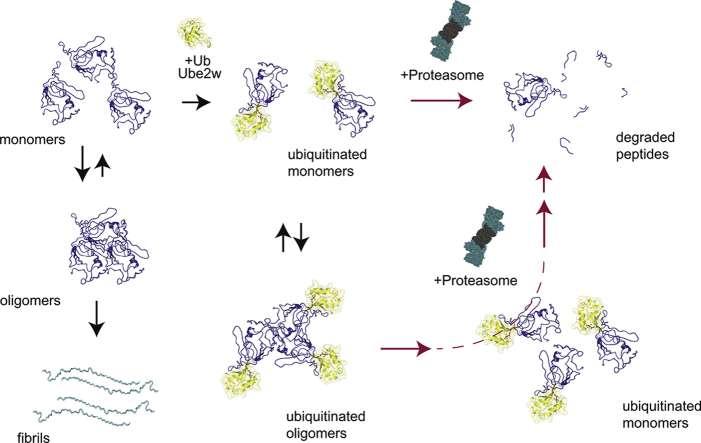

## Introduction

The 26S proteasome holoenzyme is responsible for selective protein degradation in eukaryotic cells [2]. Proteins selected for degradation are often covalently modified with ubiquitin (Ub) moieties, which are recognized by the proteasome [2]. The proteolytic activity required for degradation is provided by the 20S core particle (CP) of the holoenzyme, whereas the 19S regulatory particle (RP) that caps the CP on one or both ends is responsible for substrate recognition and ATP-dependent substrate unfolding and translocation into the CP [[3], [4], [5]]. Many biological processes are dependent on the proteasome through controlled degradation of key regulatory factors, including homeostasis, unfolded protein response, and proteostasis [6]. An important role of proteasomes is to degrade damaged proteins, thereby preventing the accumulation of misfolded and amyloidogenic proteins, which have a propensity to form aggregates [7].

Aggregation of amyloidogenic proteins progresses through several stages, during which protein monomers assemble into soluble aggregates (oligomers) that through further aggregation events eventually undergo conformational rearrangement into filamentous aggregates (fibrils). The process of protein aggregation is harmful to normal cell physiology and is often associated with neurodegenerative disorders [8]. At the cellular level, accumulation of aggregates could be due to an increased rate of aggregation or decreased rate of aggregate removal, due to, e.g., changes in the ability to disassemble or degrade aggregates. Aggregates assembled from amyloidogenic proteins tau and α-synuclein (αS) have been implicated in Alzheimer disease (AD) and Parkinson disease (PD), respectively [9,10]. Both tau and αS are intrinsically disordered in their nonamyloid state as monomers and have been reported to be degradation-resistant as aggregates [[11], [12], [13], [14]].

The inability to process certain aggregates may be coincident with proteasome malfunction, which in certain brain regions of AD and PD patients have been reported with decreased activity [15,16]. We recently demonstrated that the mammalian proteasome holoenzyme possessed a fibril-fragmenting activity, reducing the size of large tau and αS fibrils into smaller entities *in vitro* [1]. Importantly, the proteasome catalyzed this fibril-fragmenting process in a Ub-independent manner. It is currently unclear how these smaller aggregate entities may be further processed by the cellular mechanisms. A recent study has further detailed the interactions of small soluble aggregated amyloidogenic proteins (oligomers) with the proteasome, which is markedly impaired by oligomer binding [17].

Studies in cells have indicated that monomeric tau and αS proteins could be degraded by the proteasome in a Ub-dependent manner [[18], [19], [20], [21]], suggesting that aggregates of ubiquitinated proteins may accumulate when proteasomal functions are compromised. This assumption is supported by the observation of abundantly monoubiquitinated tau fibrils isolated from AD patient brain samples [22]. In addition, αS in the PD-associated Lewy bodies is also mainly monoubiquitinated [16,23]. Both tau and αS have dedicated Ub ligases, AXOT/MARCH7 [24] and SIAH1 [25,26], respectively, which preferentially monoubiquitinate their substrates. UBE2W, a Ub-conjugating enzyme that directly monoubiquitinates the N-terminus of intrinsically disordered proteins [27], has also been shown to modify tau [22,23]. Such N-terminal monoubiquitination is a well-defined degron recognized by the Ub-fusion degradation (UFD) pathway, which has been found in both yeast [28] and mammalian systems [29,30] to target misfolded proteins for proteasomal degradation and prevent cell stress. It is plausible to further hypothesize that aggregates assembled from N-terminal Ub-modified proteins would also recruit proteasomes for processing through the UFD pathway.

Here we show that the mammalian proteasome holoenzyme can target oligomers assembled from ubiquitinated tau aggregation domain (tau^K18^) and αS. We found that both tau^K18^ and αS may become ubiquitinated on the N-terminus by UBE2W. Using genetically engineered proteins with an N-terminal Ub moiety on tau^K18^ and αS, we demonstrated that such Ub modification delayed the aggregation process, which resulted in distinct aggregate structures compared with their unmodified counterparts. In addition, proteasomal functions were maintained in the presence of these Ub-modified aggregates. This was supported by data from single-molecule fluorescence spectroscopy experiments, which found a reduction in the number and the size of oligomers following proteasome treatment. The ability to target oligomers was not affected by Velcade-mediated inhibition of proteasomal proteolytic activity, suggesting that oligomer disassembly is not dependent on degradation. Based on these observations, we propose that N-terminal Ub modification on tau and αS enables proteasomes to target and remove oligomers assembled from these modified proteins.

## Results

### N-terminal Ub modification on αS and tau^K18^ delays protein aggregation

**Fig. 1:**
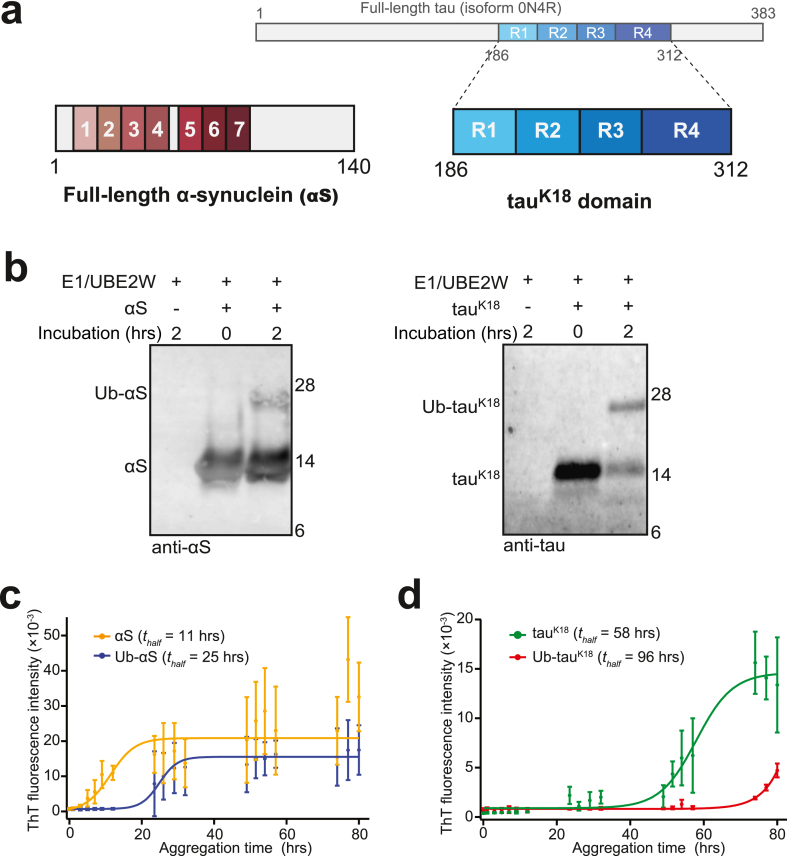
Aggregation of N-terminally Ub-modified tau and α-synuclein (αS). (a) Full-length αS (containing at seven repeats) and the tetra-repeat domain (tau^K18^) of tau. Full-length tau (isoform 0N4R) is shown on top. (b) Ubiquitination of αS (*left*) and tau^K18^ (*right*) by UBE2W. UBE2W faithfully ubiquitinated tau^K18^ and αS after 2 h incubation at 25 °C, demonstrated by the shift in the band size. Results shown are representative of reactions independently repeated at least three times. (c–d) Aggregation of unmodified and Ub-modified αS and tau^K18^, detected by ThT. (c) Ub-αS (blue) or αS (yellow) at 40 μM were aggregated under similar conditions with shaking at 37 °C. (d) Ub-tau^K18^ (red) or tau^K18^ (green) at 10 μM aggregated under similar conditions, without shaking, at 37 °C. Error bars represent standard deviation of independent triplicate measurements.

We chose to use full-length αS and a tetra-repeat domain of tau (tau^K18^) as model amyloidogenic proteins because both protein constructs have a similar molecular weight (~14 kDa, Fig. 1a). Using established protocols to purify untagged recombinant proteins of wild-type αS and tau^K18^ fragment, we found that both proteins could be ubiquitinated by UBE2W (Fig. 1b). The reaction did not continue beyond monoubiquitination as UBE2W specifically recognizes disordered sequences at the N-terminus of the substrate.

Protein ubiquitination by UBE2W did not reach completion after 2 h, resulting in a two-component mixture of Ub-modified and unmodified proteins. To obtain homogenous and pure Ub-modified αS and tau^K18^, we genetically engineered constructs that expressed fusion proteins with a single Ub moiety immediately before the first residue of αS or tau^K18^ (Ub-αS and Ub-tau^K18^, SFig. 1). These engineered N-terminal Ub-fusion proteins were protected from deubiquitination by a Gly76Ser substitution of the C-terminal residue of Ub. We further separately cloned the sequences of αS and tau^K18^ alone and purified these unmodified recombinant proteins using the same procedure as for the engineered Ub-modified proteins for consistency (see **Materials and Methods**).

Ub-αS and Ub-tau^K18^ were allowed to aggregate under similar conditions as their unmodified counterparts and measured by thioflavin T (ThT), which bound to β-sheet-rich amyloid structures. Unmodified αS entered an exponential phase reaching a half-saturated ThT intensity (*t*_*half*_) at ~11 h (Fig. 1c). In contrast, the aggregation of Ub-αS showed an extended lag phase and reached half-saturation with a delay of 14 h (*t*_*half*_ = 25 h). This Ub-dependent delay was even more apparent for Ub-tau^K18^ (estimated *t*_*half*_ of 96 h), whose aggregation was delayed by ~38 h compared with tau^K18^ (*t*_*half*_ = 58 h, Fig. 1d). These results suggest that Ub modification might decrease the rate of aggregate formation and/or the level of total amyloid aggregates under our reaction conditions.

**Fig. 2:**
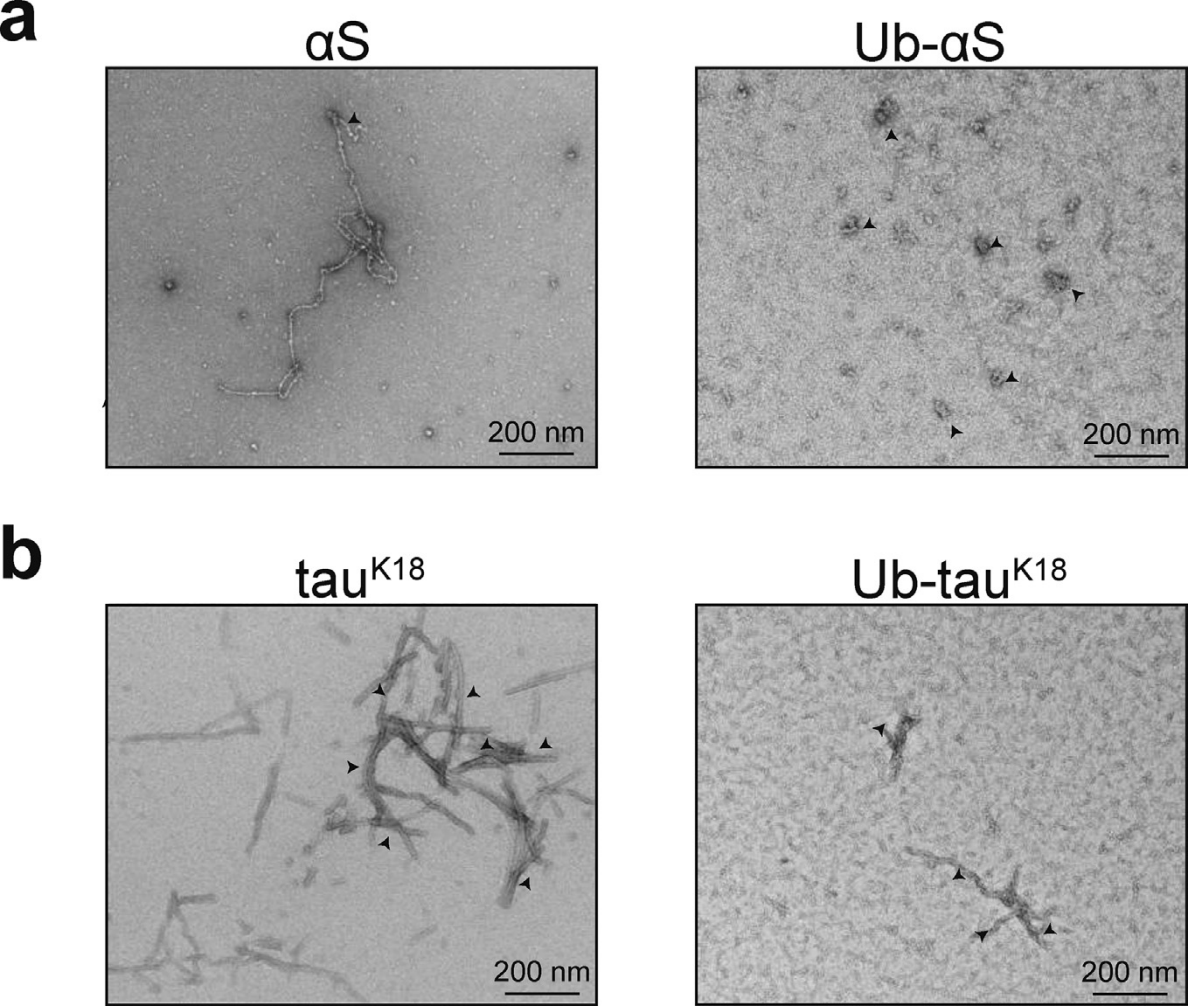
Detection of aggregates by transmission electron microscopy (TEM). (a) Filamentous or amorphous aggregates assembled from αS (*left*) and Ub-αS (*right*) after 96 h of aggregation reaction. Arrows highlight positions of some typical aggregate structures. (b) Filamentous aggregates of tau^K18^ (*left*) and Ub-tau^K18^ (*right*) detected by TEM as in (a).

Aggregates assembled beyond 96 h were further imaged under TEM to qualitatively compare the effect of Ub modification. Interestingly, although fibrils were detected from unmodified αS, those formed from Ub-αS mostly appeared as small amorphous assemblies (Fig. 2a). Despite repeated attempts, we could not detect any filamentous aggregates from Ub-αS under TEM. In comparison, Ub-modified tau^K18^ assembled into aggregates that appeared thinner and less filamentous-like than unmodified tau^K18^, which were detected abundantly (Fig. 2b). These results indicate that the morphology of filamentous aggregates is affected by N-terminal Ub modification.

### Single-molecule measurements of Ub-modified aggregates

**Fig. 3:**
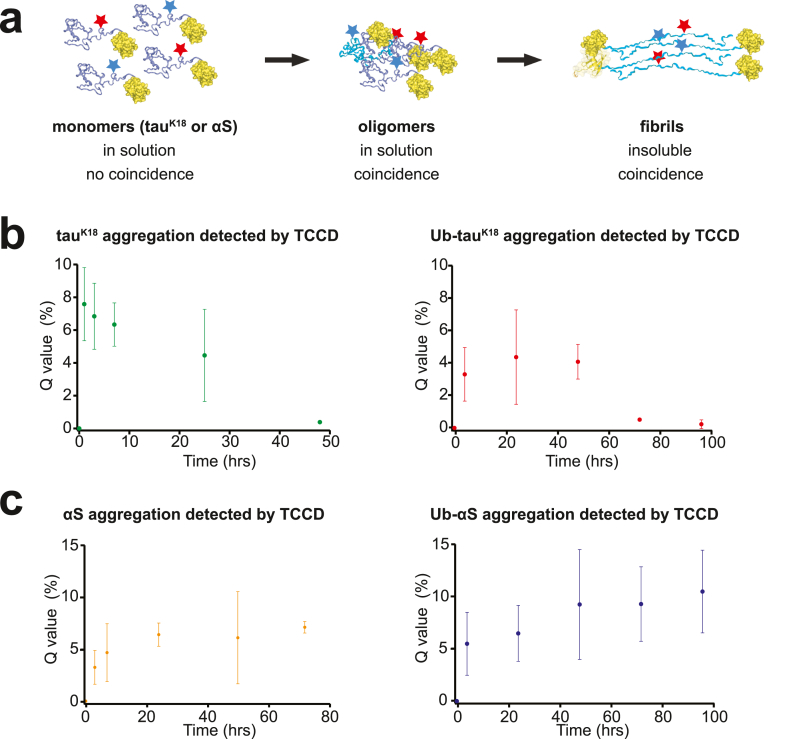
Single-molecule fluorescence detection of tau^K18^ and αS aggregates. (a) Schematic representation of aggregation from N-terminally Ub-modified amyloidogenic proteins (in blue; Ub in yellow) tagged with Alexa488 (marine stars) and Alexa647 (red stars) in a 1:1 stochiometric ratio. Monomeric proteins (*left*) carry a single fluorescent dye and cannot be detected using the coincidence criterion **(Materials and Methods)**. Soluble oligomers (*middle*) will carry both dyes and satisfy the coincidence criterion. As aggregation progresses, fibrillar aggregates (in cyan; *right*) will form that may be insoluble or too large for detection. (b) Aggregation of tau^K18^ (*left*) or Ub-tau^K18^ (*right*) as detected by single-molecule measurements. The *Q* value is proportional to the percentage of oligomers. Error bars represent standard deviation of three independent measurements. (c) Aggregation of αS (*left*) or Ub-αS (*right*) detected by single-molecule measurements as in (b).

We previously established single-molecule fluorescence methods to measure the aggregation process independently of aggregate structure and to estimate the proportion of soluble aggregates (oligomers) [[31], [32], [33]]. In this study, we applied the same method and labeling strategy to attach fluorescent dyes to αS and tau^K18^ (SFig. 2a). Using this approach, we mixed the same protein labeled with Alexa488 or Alexa647 in a 1:1 stochiometric ratio and initiated the aggregation reaction. Aggregate samples were flowed through a microfluidic channel and excited at suitable wavelengths with two overlapping lasers using a confocal microscope (SFig. 2b). Oligomers (here defined as 2-150mers) formed during aggregation will contain both dyes and give rise to coincident fluorescent bursts when they pass through the confocal volume of the laser (two color coincidence detection (TCCD)) [33], whereas any monomer signal will not give rise to coincident fluorescent bursts (Fig. 3a). The fraction of all fluorescence bursts that are coincident is proportional to the fraction of oligomers present and is measured by the *association quotient Q* (see **Materials and Methods**).

We could reproducibly detect oligomers from both tau^K18^ and Ub-tau^K18^ early in the aggregation process (Fig. 3b). The presence of tau^K18^ oligomers remained steady within the first 24 h, qualitatively consistent with our previously published results [32]. In comparison, Ub-tau^K18^ oligomers could be detected at a steady level up to 48 h from the start of aggregation reaction. An apparent reduction of the fraction of soluble oligomers with time was detected in both unmodified and Ub-modified tau^K18^ beyond 50 and 70 h, respectively. The loss of soluble oligomers as aggregation progresses had previously also been observed for tau^K18^ without Ub modification [32] and is likely due to the presence of aggregates that are either insoluble or too large to enter the microfluidic channel, hence not detected by single-molecule TCCD. The decrease in the calculated *Q* values were coincident with a reduction in the overall fluorescence signal measured, as the total number of dye labels passing through the volume has decreased, further supporting insoluble fibril formation.

A steady state population of soluble oligomers appeared in the aggregation of both unmodified and Ub-modified αS and did not change appreciably as the reaction proceeded over longer incubation times (Fig. 3c). Only a fraction of aggregates is therefore able to form ThT-active aggregate species, where the formation of Ub-modified αS aggregates is delayed compared with unmodified αS (shown in Fig. 1c). The ThT assays further suggest that there could be more β-sheet content in the aggregates formed from unmodified αS, as bulky Ub moieties may obstruct close packing of Ub-αS aggregates.

### Proteasomes are able to target Ub-modified aggregates

To study the degradation of Ub-modified amyloidogenic proteins, we purified proteasome holoenzyme from an established mammalian cell line [34] using an affinity column (SFig. 3a). The purified proteasome holoenzyme was resolved and validated by SDS-PAGE (SFig. 3b), transmission electron microscopy (TEM, SFig. 3c) and native gel electrophoresis in the presence of an ATP-containing buffer (SFig. 3d). We could not quantitatively detect the presence of free proteasomal CPs with Coomassie staining or under TEM. A batch of the yeast proteasome holoenzyme was used as the molecular weight reference for the detection of capped and uncapped CP under similar conditions (SFig. 3e). All four Dye-labeled tau^K18^ and αS protein constructs could be quantitatively degraded by the proteasome, confirming their activity (SFig. 4a and b).

It has been reported that nonubiquitinated aggregates resist degradation [[11], [12], [13], [14]], and both tau aggregates and αS oligomers have recently been shown to impede proteasome activity [[13], [17]]. In addition, we recently demonstrated that the product of proteasome-catalyzed fragmenting of tau and αS fibrils was small aggregate entities, supporting our previous report that proteasomes had no effect on soluble αS oligomers without ubiquitin modification [39]. We therefore tested whether N-terminal Ub modification on tau and αS oligomers would enable their disassembly by proteasomes. ThT was found to bind to the proteasome, which interfered with fluorescence measurements (SFig. 4c). Turning to single-molecule TCCD approach, we reproducibly detected a decrease in the level of soluble oligomers in both Ub-tau^K18^ and Ub-αS as late as 96 h into the aggregation reaction (Table 1 shows a typical experiment). Ub-tau^K18^ oligomers generated throughout the assembly process were largely removed after incubation with the proteasome (Table 1a). The effect was also significant for oligomers assembled from Ub-αS, which decreased in the presence of the proteasome (Table 1b).

**Table 1:** Representative TCCD experiments of proteasomal degradation of (a) Ub-tau^K18^ and (b) Ub-αS aggregates, respectively, illustrating how the single-molecule data were analyzed (see STable 1 for aggregate size dependency analysis). The experiments were performed in triplicate (see Fig. 4 for average results of repeat experiments). At the indicated times after aggregation initiation (first column), the number of aggregates were counted after incubation with either the control buffer (second column) or with the proteasome (third column). The calculated percentage reduction of aggregates is shown (fourth column).

a. Proteasomes remove Ub-tau^K18^ oligomers			
Aggregation time	Number of oligomers control	Number of oligomers + proteasome	Oligomer loss
4	1053	57	95%
24	9802	561	94%
48	5670	318	94%
72	2880	1676	42%
96	278	98	65%

b. Proteasomes remove Ub-αS oligomers			
Aggregation time	Number of oligomers control	Number of oligomers + proteasome	Oligomer loss
4	6480	1582	76%
24	2930	660	77%
48	2158	840	61%
72	1737	946	46%
96	3726	1875	50%

We previously used the relative fluorescence intensities of aggregates compared with monomers to calculate the apparent size of aggregates [31]. Analyzing the data in Table 1 revealed that the proteasome caused a general decrease in the aggregate level independently of the apparent aggregate size (STable 1), suggestive of gradual aggregate disassembly.

**Fig. 4:**
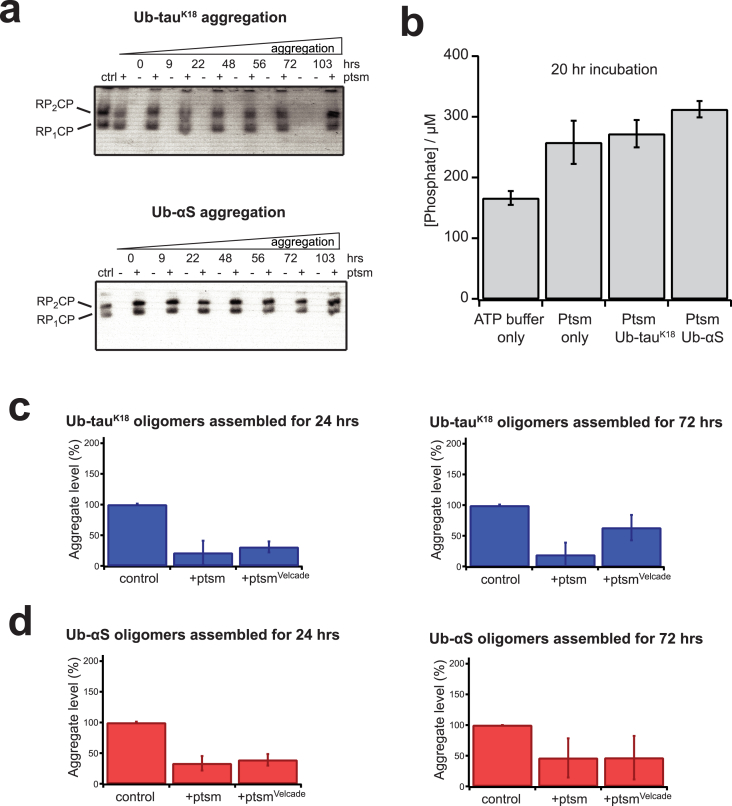
Proteasome maintains its function in the presence of Ub-modified oligomers, which are disassembled independently of the proteolytic activity. (a) Ub-tau^K18^ (*top*) or Ub-αS (*bottom*) aliquots from indicated aggregation times were incubated with the proteasome or with control buffer. After reaction completion, samples were resolved on a 3% native gel and visualized by LLVY-AMC fluorescence emission (*λ*_*Ex*_ = 340 nm, *λ*_*Em*_ = 440 nm). A sample of the proteasome at the same concentration alone was used as control (ctrl). (b) Phosphate assay reporting the concentration of free phosphates after incubating proteasomes with Ub-modified oligomers for 20 h. Residual free phosphates were present in the ATP-containing buffer, but increased in presence of the proteasome. The free phosphate levels were not reduced when the proteasome was incubated with Ub-modified tau^K18^ or αS, suggesting that ATP hydrolysis was not affected. (c–d) Velcade does not affect aggregate removal by the proteasome. (c) Ub-tau^K18^ and (d) Ub-αS oligomers were assembled for 24 h (*left*) or 72 h (*right*) before mixing with the prepared proteasomes, incubated and subsequently measured by single-molecule TCCD. Proteasomes were preincubated with Velcade for 5 min before mixing with substrates. The percentage change in aggregate level is relative to the control sample without proteasome. Error bars represent standard error of mean from three sets of independent measurements.

Our previous work using the same confocal single-molecule technique found that the proteasome did not target oligomers that are not modified with Ub [11] and that these oligomers are not affected by active chaperones [35]. We therefore attribute the observed reduction in aggregate level specifically to the proteasome. No change in the proteasomal proteolytic or the ATP-dependent activity after incubation with Ub-modified oligomers was observed (Fig. 4a and b), consistent with observations after incubating proteasomes with fibrils [1].

The fibril-fragmenting activity of the proteasome was shown to be independent of the peptidase activity [1]. To test whether the disassembly of Ub-modified oligomers also did not rely on the proteasomal peptidase activity, we repeated the single-molecule experiments using proteasomes that were preincubated with Velcade as in [1]. The decrease in Ub-tau^K18^ or Ub-αS oligomers was not affected by Velcade-mediated inhibition (Fig. 4c and d), consistent with our recent observation [1].

The ability of proteasomes to target Ub-modified oligomers was further resolved by Western blotting. Oligomers assembled from both Ub-tau^K18^ and Ub-αS were prone to proteasomal activity (SFig. 5a and c), whereas oligomers assembled from unmodified tau^K18^ and αS showed no detectable intensity change in the presence or absence of proteasome treatment (SFig. 5b and d). This is consistent with the hypothesis that oligomers formed from proteins N-terminally modified with Ub can be targeted by the proteasome, whereas unmodified oligomers remain unchanged.

## Discussion

Our current work has shown that the presence of an N-terminal Ub moiety on tau^K18^ and αS results in distinct aggregation kinetics and aggregate conformations. Oligomers assembled from these Ub-modified proteins are prone to proteasomal activity. We previously demonstrated that proteasomes are able to target fibrils, as they are structurally distinct from nonfilamentous aggregates. This study further complements our previous demonstration of proteasomal fibril-fragmenting activity [1] with an additional proteasomal ability to target Ub-modified oligomers. Intriguingly, size analysis of oligomers at various times of aggregation did not indicate any clear trends in their susceptibility to be removed by proteasomes (STable 1), possibly suggesting that the gradual disassembly of oligomers may not be a straightforward process.

As tight assembly and packing are key features of amyloid aggregates, presence of a Ub moiety at the N-terminus could potentially induce soluble oligomers into a conformation distinct from unmodified protein aggregates. In another of our previous work, we found that arachidonic acid could induce a conformational change in soluble αS oligomers that could subsequently be targeted by the proteasome [11], suggesting that the proteasome acted more effectively on induced than uninduced oligomers, which contained more compact structures.

N-terminal monoubiquitination is associated with enzymes of the UFD pathway, which have been found in mammalian systems to target misfolded proteins [29,30] and foreign particles [36] for proteasomal degradation to prevent cell stress. Our current study demonstrates that such N-terminally monoubiquitinated proteins may assemble into soluble oligomers, which are distinct to aggregates assembled from proteins ubiquitinated on Lys residues [25,26,[37], [38], [39]]. As demonstrated here, N-terminally modified oligomers may be efficient proteasomal targets that exist transiently in cells and could therefore have escaped detection in traditional mass spectrometry analyses. The development of antibodies specific for N-terminal monoubiquitination [36] and a dedicated mass spectrometry proteomics approach [40] will likely provide additional insights into this unique ubiquitination type.

The complete monoUb modification on every protein within each oligomer in our study may provide avid recognition *in vitro* and enhance their disassembly efficacy at the proteasome. Physiologically, ubiquitination levels of tau and αS are likely to be dynamically regulated through both protein synthesis and degradation. Ub modifications on different positions within the proteins have distinct consequences for their aggregation properties. Our current study proves a novel concept of how N-terminal Ub modification impedes aggregation, enables proteasomes to target oligomers, and provides a possible option for oligomer disassembly by the ubiquitin-proteasome system.

## Material and Methods

### Molecular biology of plasmids for protein expression

A single Ub moiety was expressed in tandem to the protein of interest (tau^K18^ or αS). The DNA sequence of Ub was introduced either at the 5′ end, immediately before the ATG codon. When the Ub coding sequence was cloned upstream of the wild-type αS or tau^K18^ coding sequences, a mutation corresponding to Gly to Ser was introduced at residue 76, the last residue of Ub. The constructs were subsequently subcloned into a pOPINF vector using restriction enzymes, resulting in a His_6_-tag at the N-terminus of the Ub. Tau^K18^ or αS sequences were also separately cloned into the pOPINF vector without the Ub sequence, so that the His_6_-tag is immediately N-terminal to the substrate.

Cys mutations were introduced using site-directed mutagenesis on Ala90 of αS or Ile202 of tau (annotation based on the 0N4R isoform sequence). We previously showed that introduction of Cys and subsequent dye-labeling did not disrupt the integrity and aggregation properties of these proteins [31,32]. Equivalent mutations were separately introduced into Ub-αS and Ub-tau^K18^ constructs. The two other Cys residues in wild-type tau^K18^ sequence were mutated to Ala [32].

The full-length sequence of mouse E1 in pET28a vector (kind gift from David Komander) and human UBE2W in pET15b vector (kind gift from Wade Harper, Addgene plasmid #15809) were used for protein expression. Plasmids for protein expression of full-length αS in pT7-7 vector or tau^K18^ sequence in pJExpress vector (custom designed) alone coded for untagged constructs of the wild-type sequence of human αS or tau^K18^. These untagged constructs coded for wild-type sequences of the N-terminal residues used in the ubiquitination assay by UBE2W.

### Recombinant protein purification

Plasmids were transformed into Rosetta2 (DE3) pLysS cells (Novagen) and grown in LB media to OD_600_ = 1.0 before overnight induction with 1 mM IPTG at 20 °C. Cells were collected the next day by centrifugation at 5000 × *g* for 30 min before lysis by sonication. The cell lysate was cleared by centrifugation at 21,000 × *g* for 30 min at 4 °C.

The supernatant from purification of His_6_-tagged proteins was loaded onto a self-packed cobalt column (Clontech). Unbound proteins were washed off with Loading Buffer (50 mM Tris–HCl [pH 7.4], 100 mM NaCl, 10 mM imidazole), and bound proteins subsequently eluted with Elution Buffer (50 mM Tris–HCl, 100 mM NaCl, 200 mM imidazole, pH adjusted to 7.4).

Preparation of the supernatant from purification of untagged αS and tau^K18^ followed established protocols (e.g. Refs. [31,41]). In brief, the cleared supernatant was poured into a 50-ml falcon tube and incubated in boiling water for 10 min before cooling down to room temperature. The solution was cleared with a second centrifugation step at 21,000 × *g* for 30 min at 4 °C, and the supernatant was filtered before further purification.

The eluted or filtered samples were further purified using ion exchange (IEX) chromatography columns HiTrapQ (for αS constructs) or HiTrapS (for tau constructs), running a linear NaCl gradient from Buffer A (50 mM Tris–HCl [pH 7.4], 50 mM NaCl) up to 1 M NaCl.

Peak fractions from the IEX were concentrated to <4 ml and loaded onto a Superdex 16/60 gel filtration column (GE Healthcare) in Buffer A. Eluted fractions were separated by SDS-PAGE, and fractions judged to be >99% pure by Coomassie stain were further concentrated and flash frozen in aliquots (SFig. 1). Protein concentrations were measured on a NanoDrop.

### Dye-labeling on proteins

Proteins carrying Cys substitutions were dialyzed into Labeling Buffer (50 mM Tris–HCl [pH 7.2]) before dye-labeling. AlexaFluor 488 C5 maleimide or AlexaFluor 647 C2 maleimide (Invitrogen) were dissolved in DMSO and added to the proteins in a 1:1.2 stochiometric ratio of excess dye. We routinely use this protocol to ensure that essentially all proteins are labeled as detected by ion exchange chromatography, size exclusion chromatography, and mass spectroscopy. The labeling reaction was quenched after 1 h with fresh DTT at 100 mM final concentration and loaded onto a HiPrep 26/10 desalting column (GE Healthcare). The final labeled proteins were concentrated to at least 80 μM and flash frozen in Protein Buffer (50 mM Tris–HCl [pH 7.2], 50 mM NaCl, 0.01% Tween20) in small aliquots. Concentrations were determined by NanoDrop.

### Purification of mammalian proteasomes

Proteasomes were purified from a HEK293T cell line stably expressing an Rpn11-TEV-Biotin tag (kind gift of Lan Huang, UC Irvine) using established protocols [34]. Briefly, cells were grown to 100% confluence and collected with a scraper before resuspension in ice-cold Proteasome Buffer (50 mM Tris [pH 7.5], 0.5% NP-40, 10% glycerol, 5 mM ATP, 1 mM DTT, 5 mM MgCl_2_). A Dounce homogenizer was then used to lyse the cells, and the lysate was cleared by centrifugation at 3000 × *g* for 5 min at 4 °C. The lysate was incubated overnight at 4 °C in 2-ml bed volume of preequilibrated NeutrAvidin resin beads (Pierce). Unbound proteins were washed off with Proteasome Buffer. Proteasome was subsequently released from the column with TEV protease (Invitrogen) at 30 °C and concentrated to > 2 μM before flash freezing.

### UBE2W ubiquitination assays

Ubiquitination assays were carried out at 25 °C in Ubiquitination Buffer (50 mM Tris [pH 7.5], 10 mM ATP, 1 mM DTT, 10 mM MgCl_2_), containing 0.5 μM of E1, 1 μM of UBE2W, 200 of μM wild-type Ub (Sigma), and 10 μM of untagged αS or tau^K18^ substrate. The reactions were incubated for 2 h before quenching by Laemmli Buffer containing reducing agent. Substrates were excluded in control samples to test for cross-reactivity of the antibodies used with the ubiquitinating enzymes or the Ub (Fig. 1b).

### Protein aggregation assays

For protein aggregation, Ub-tau^K18^ or tau^K18^ were diluted in PBS Buffer (MP Biomedicals) containing 0.01% sodium azide to 10 μM final concentration and incubated at 37 °C. An equimolar amount of heparin (H3393, Sigma-Aldrich) was added to initiate tau^K18^ aggregation reactions. Aggregation assays for Ub-αS or αS were performed at 40 μM final protein concentration in PBS Buffer containing 0.01% sodium azide and incubated at 37 °C shaking, as described [31]. We did not detect pellets of insoluble fibrils after 10 min centrifugation at 13,000 × *g* for Ub-tau^K18^ or Ub-αS aggregates.

### Thioflavin T fluorescence assays

Thioflavin T (Sigma) was dissolved in PBS and filtered through a 0.02 μm filter. The concentration was determined by UV absorbance at 405 nm on a NanoDrop. Aliquots were removed from tau^K18^ (10 μl) or αS (5 μl) samples at indicated times after aggregation initiation and mixed with 40 μl of the ThT solution at 10 μM. The mixture was incubated for 10 min and subsequently measured on a spectrophotometer (*λ*_*Ex*_ = 415 nm, Varian Eclipse). Integral area between 460 and 560 nm of the emission spectrum was calculated for each time point. The mean value of triplicate aggregation assays was used to plot Fig. 1c and d. Each data set was fitted to a sigmoidal function, defined as

(1) $$Intensity_{ThT}=\frac{1}{1+e^{t}}$$

where *t* is the time after aggregation initiation in hrs and *Intensity*_*ThT*_ is the mean integral area of fluorescence emission. All plots were calculated and generated using IgorPro (Wavemetrics). The sigmoidal behavior of aggregation produces the time, *t*_*half*_, needed for *Intensity*_*ThT*_ to reach 50% of the maximal plateau value.

### Degradation assays

Degradation assays were typically performed at 25 °C in Degradation Buffer (50 mM Tris–HCl [pH 7.5], 10 mM ATP–MgCl_2_, 30 mM creatine phosphate, 4 μM creatine kinase) containing 40 nM proteasome. Control samples were set up in the same buffer without adding the proteasome. Tau^K18^ substrates were reacted for 3 h at a final concentration of 2.6 μM in PAGE-based assays and 0.2 μM in single-molecule assays. Incubation time for αS samples was 12 h and diluted to a final concentration of 10 μM in PAGE-based assays and 1 μM in single-molecule assays. We occasionally observe secondary dimer or higher bands emerging with prolonged incubation when the substrate concentration is higher than 10 μM, indicating sporadic aggregation during degradation assays. For the inhibitor assays, the proteasome was preincubated for 5 min at 25 °C with Velcade (Proteasome^Velcade^) to a final concentration of 100 μM.

### Resolving of aggregate samples by gel electrophoresis and Western blotting

Proteasomes used in the overnight degradation assays were resolved on self-poured 3% polyacrylamide native gels and detected with a fluorogenic model substrate, LLVY-AMC, as described previously [42]. For Western blotting, protein samples were first separated on 4–12% NuPAGE gels (Invitrogen) and then transferred to PVDF membranes as per manufacturer's protocol (Mini Trans-Blot wet transfer, Biorad). Samples taken were quenched with Lammeli buffer but not heated (to preserve the oligomeric bands). Mouse anti-tau (1E1/A6, Merck) or rabbit anti-αS (ab138501, Abcam) were used as primary antibodies following standard Western blotting methods. Secondary anti-mouse and anti-rabbit antibodies compatible with detection on an Odyssey CLx Imager or a Typhoon scanner were purchased from Li-Cor or Invitrogen.

### Colorimetric phosphate assay

ATPase kit containing malachite green and ammonium molybdate was purchased from Abcam (ab65622). For assays in Figs. 4b, 40 μl of each reaction (set up as described in **Degradation assays** section) was mixed with 6 μl of the malachite green reagent and incubated for 15 min before measurement. The colorimetric output was measured at OD = 650 nm on a microplate reader. A linear standard curve from 0 to 20 mM of free phosphates was established to convert colorimetric reading into phosphate concentration. Three independent replicate experiments were performed for each reaction.

### Transmission electron microscopy imaging

Samples shown in Fig. 2 were aggregated beyond 96 h and applied onto a carbon-coated 400 mesh copper grid (Agar Scientific). Mammalian proteasome was applied at 100 nM concentration. The grids were then washed with double distilled water and stained with 2% (w/v) uranyl acetate for 1 min. TEM images were acquired using Tecnai G2 microscope (13218, EDAX, AMETEK) operating at an excitation voltage of 200 kV.

### Single-molecule measurements

The instrument setup and collection of single-molecule data are based on our earlier works and have been extensively described (e.g., Refs. [31,43]). Briefly, single-molecule data were collected on a custom-built system using overlapping lasers with excitation maxima at 485 nm and 640 nm (see SFig. 2a). The rate of modulation for both lasers was at 10 modulations per millisecond. Samples were measured under flow using custom-made PDMS microfluidics devices following published procedures [45]. Aggregates were assembled from protein monomers labeled with either AlexaFluor 488 or AlexaFluor 647 and mixed in a 1:1 stochiometric ratio for aggregation (SFig. 2a). This ensures that only aggregates will carry both dyes and will be detected by the coincidence criterion [33].

Degradation assays were performed for 3 h (Ub-tau^K18^) or 12 h (Ub-αS) and diluted for immediate single-molecule measurement. Fluorescent protein samples were diluted to 100 pM (Ub-αS) or 40 pM (Ub-tau^K18^) final concentration and measured under flow according to previously established methods [32,44]. The bin time and flow rates for Ub-αS and Ub-tau^K18^ constructs were individually optimized to achieve the highest value of *Q* as described previously [11,32]. Ub-αS aggregates were measured at 100 μl/h flow rate, and the fluorescence signals were collected with 0.1 ms bin time. For Ub-tau^K18^ samples, the flow rate and bin time were 50 μl/h and 0.2 ms, respectively. Data were typically collected for 15 min at 25 °C in frames of 50,000 bins. Independent triplicate experiments were performed for Ub-tau^K18^ and Ub-αS aggregation reactions. A representative set of aggregate degradation measurements is shown in Table 1.

### Single-molecule data analysis

We used the AND criterion to detect coincidence events in the two channels [33]. This separates aggregation events from background monomers by accepting only those signals for which the blue- and the red-excited channels are above the threshold value. The proportion of monomers that are associated to form oligomers is expressed using the association quotient *Q*, which is defined as

(2) $$\text{Q}=\frac{\text{C}-\text{ABτ}}{\text{A}+\text{B}-\left(\text{C}-\text{ABτ}\right)}\times 100\%$$

where A and B are the rates of detection of events in the two fluorescent channels, respectively, and C is the detection rate of coincident events; τ is the interval time of detection so that the ABτ expresses the coincident events that occur by chance [33]. We also measured the level of background detection of the buffer containing no fluorescent proteins and applied a uniform threshold fivefold over the background in both red- and blue-detector channels (10 kHz) to remove noise signals. We found, in a previous work using this method, good agreement between size distribution of particles immobilized onto a surface and those measured in solution of the same particles [44], showing that potential laser beam inhomogeneity will not become a significant effect in these experiments. A reference duplex DNA sample of 40 base pairs labeled with AlexaFluor 488 and AlexaFluor 647 at the 5′ end of each of the single DNA strands, repeatedly gave a *Q* value of 30%, using the same analysis [43].

Each single-molecule measurement was normalized to a standard number of frames, and the number of significant coincident events was counted. The percentage decrease in oligomers upon proteasome treatment is calculated as follows:

(3) $$decrease=\frac{aggregate_{ctrl}-aggregate_{ptsm}}{aggregate_{ctrl}}$$

which is expressed in percentage. The estimation of *apparent aggregate size* was carried out as previously described [31], based on the fluorescence intensities of each aggregate as it passed through the probe volume. In brief, the approximate monomer number per aggregate can be extracted assuming that 50% of monomers are donors using the equation

(4) $$Aggregate\;size=\;\frac{I_{DA}+I_{A}/\gamma }{I_{D\_monomer}}$$

where *I*_*DA*_ represents the donor fluorescence intensity in presence of acceptor, *I*_*A*_ the acceptor fluorescence intensity. *I*_*D_monomer*_ corresponds to the average intensity of donor monomers, and γ to a correction factor that accounts for different quantum yields and detection efficiencies of the donor and acceptor.

Previous control experiments on αS aggregates showed that this analysis recovered the same apparent size distribution as that measured when the aggregates were immobilized on a glass surface [44]. Depending on the calculated apparent aggregate size, each aggregate was subsequently arbitrarily classified either as *small* (size ≤ 15), *medium* (size 16–30), or *large* (size 31–45), and the frequency of each group reported as in STable 1.

## Appendix A Supplementary data

**SFig. 1**. Purification of Ub-tau^K18^ and Ub-αS. (a) Peak fractions of Ub-tau^K18^ eluted from a typical run on gel filtration column were resolved on SDS-PAGE, and proteins were stained with Coomassie blue. (b) Ub-αS samples were also purified and eluted from gel filtration column as in (a). The fractions collected and used for experiments are underlined. Molecular weight markers are shown to the left of each gel.

**SFig. 2**. Single-molecule confocal measurements of oligomers. (a) Dye-labeling scheme used for Ub-tau^K18^ and Ub-αS. The proteins were genetically engineered to carry a single Cys residue that enables covalent fluorescent dye-labeling (see **Materials and Methods**). Each protein was labeled separately with Alexa488 or Alexa647 before mixing in a 1:1 stochiometric ratio for subsequent aggregation. (b) Model representation of the single-molecule confocal instrument setup used. A multiline laser source (iFLEX-Viper) with a modular single mode fiber delivery system was responsible for excitations at 488 nm and 640 nm. The two overlapping laser beams were focused using an objective with high numerical aperture into a microfluidic channel where a constant flow was applied to the protein sample (*zoomed in*). The sample was diluted to a concentration such that single molecules occupying the focal volume were simultaneously excited. Emitted fluorescence is captured using the same objective and separated from reflected excitation beam using a dichroic mirror (DRLP, dichroic long-pass filter). A second dichroic mirror splits the emission light beam again for detection in two separate APDs (avalanche photodiode detector). ALP (anti-light pollution) filters and AF (band pass) filters used are indicated.

**SFig. 3**. Purification of 26S proteasome holoenzyme from a HEK293-derived cell line. (a) Molecular representation of the proteasome holoenzyme (pdb-id 5GJQ). The 20S core particle (CP, gray) is capped at both ends with 19S regulatory particles (RP, teal). Rpn11 (magenta), a deubiquitinase, is the subunit tagged for affinity purification of the proteasome (see **Materials and Methods**). (b) The presence of the individual proteasomal subunits was detected by SDS-PAGE after purification. (c) A typical batch of proteasome holoenzyme visualized by TEM. The CP and RPs are highlighted. (d) Purified proteasome holoenzymes used in this study resolved by native gel electrophoresis and detected under UV with LLVY-AMC (*left*) or Coomassie-stained (*right*). (e) A batch of yeast 26S proteasomes resolved by native gel electrophoresis under the same conditions as in (d) and detected under UV (*left*) or stained with Coomassie (*right*).

**SFig. 4**. Studying proteasome activities on N-terminally Ub-modified proteins. (a) Dye-labeled αS (top) and Ub-αS (bottom) monomers can be degraded by the proteasome over time. Samples were resolved by SDS-PAGE, and fluorescence emission was detected on a Typhoon Imager. A lower molecular weight degradation fragment developed over time beneath the substrate bands. (b) Degradation of dye-labeled tau^K18^ (*top*) and Ub-tau^K18^ (*bottom*) monomers, performed and visualized as in (a). (c) ThT can bind to the proteasome. The proteasome incubated at 0, 40, or 120 nM with ThT at 10 μM final concentration. Samples were excited at 415 nm and fluorescence emission was detected from 460 to 560 nm.

**SFig. 5**. Degradation assays of Ub-modified oligomers. Samples were resolved by SDS-PAGE. Oligomers assembled from (a) Ub-tau^K18^ or (b) tau^K18^ were incubated with the proteasome or the control buffer, resolved by SDS-PAGE and transferred to a PVDF membrane for Western blot detection at high (*top*) and normal exposure (*bottom*) to detect changes in the oligomer or monomer bands. Oligomers assembled from (c) Ub-αS or (d) αS were subjected to proteasomal degradation and presented as in (a and b). Note that the proteasome alone did not contribute to any bands detected by either anti-tau or anti-αS antibodies. The reduction in oligomer bands of Ub-tau^K18^ and Ub-αS aggregates throughout the aggregation process was not an effect of the reducing agent or other factors because samples in control lanes and proteasome-containing lanes underwent the same procedure and were kept in the same buffer. Each assay was independently repeated at least three times.

**Supplementary Table 1.** Changes in oligomer size distribution following proteasomal degradation calculated from data in Table 1. (a) Ub-tau^K18^ and (b) Ub-synuclein oligomers are grouped according to their *apparent oligomer sizes* into ‘*Small*’ (15mers or smaller), ‘*Medium*’ (between 15mers and 30mers), and ‘*Large*’ (between 31mers and 45mers).
