# Supplementary material for: N-Terminal Ubiquitination of Amyloidogenic Proteins Triggers Removal of Their Oligomers by the Proteasome Holoenzyme
Source: J Mol Biol. 2020 Jan 17;432(2):585–96. doi: 10.1016/j.jmb.2019.08.021 (PMC6990400; doi:10.1016/j.jmb.2019.08.021)
Supplement: Supplementary file 1 [file mmc1.pdf]

Supplementary Figure 1

a

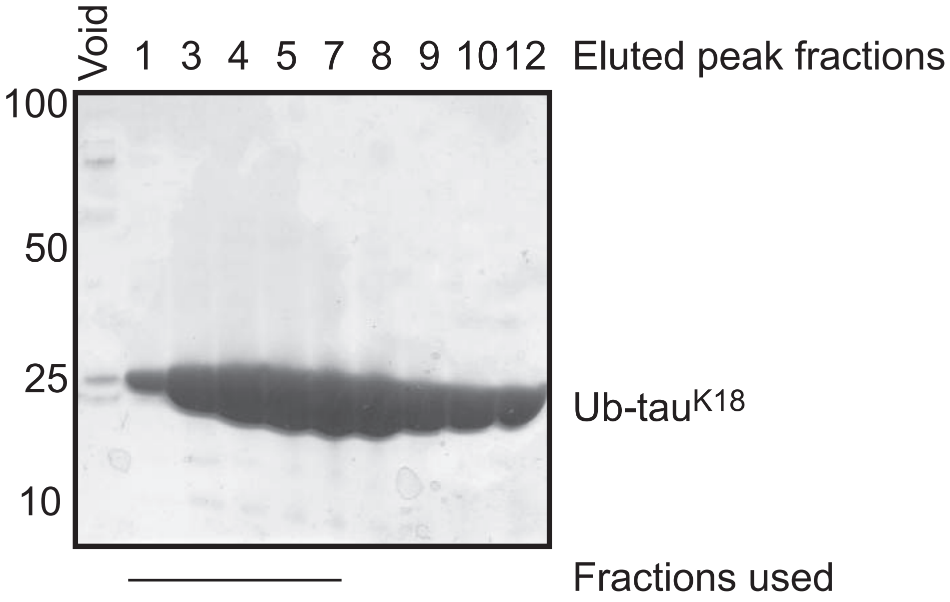

b

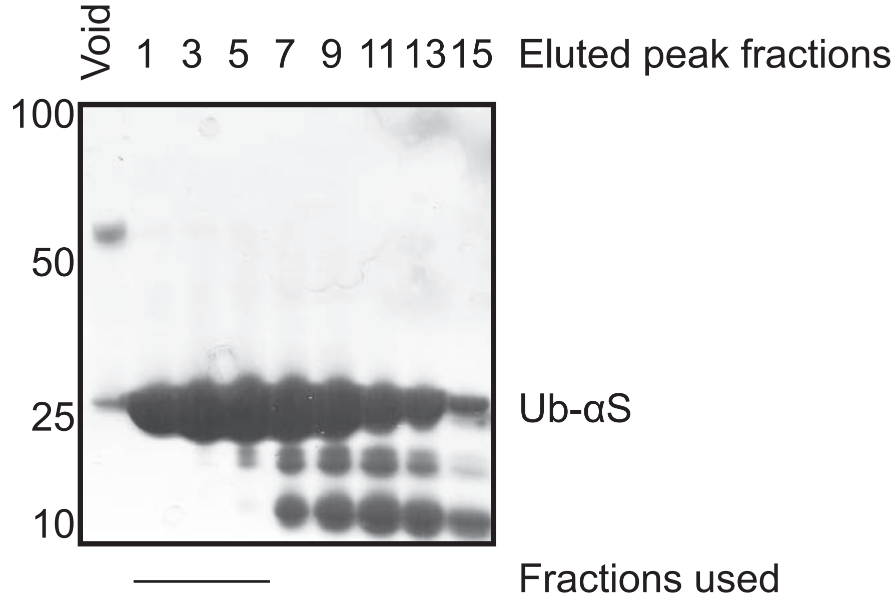

Supplementary Figure 2

a

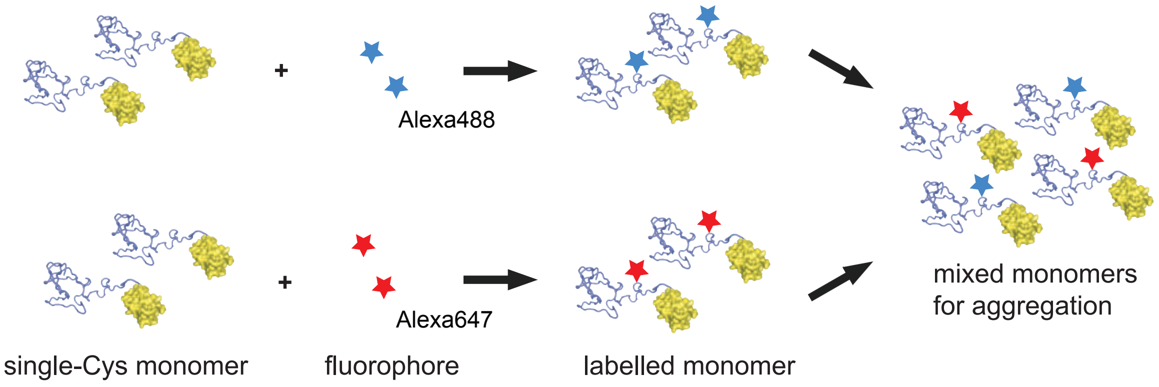

b

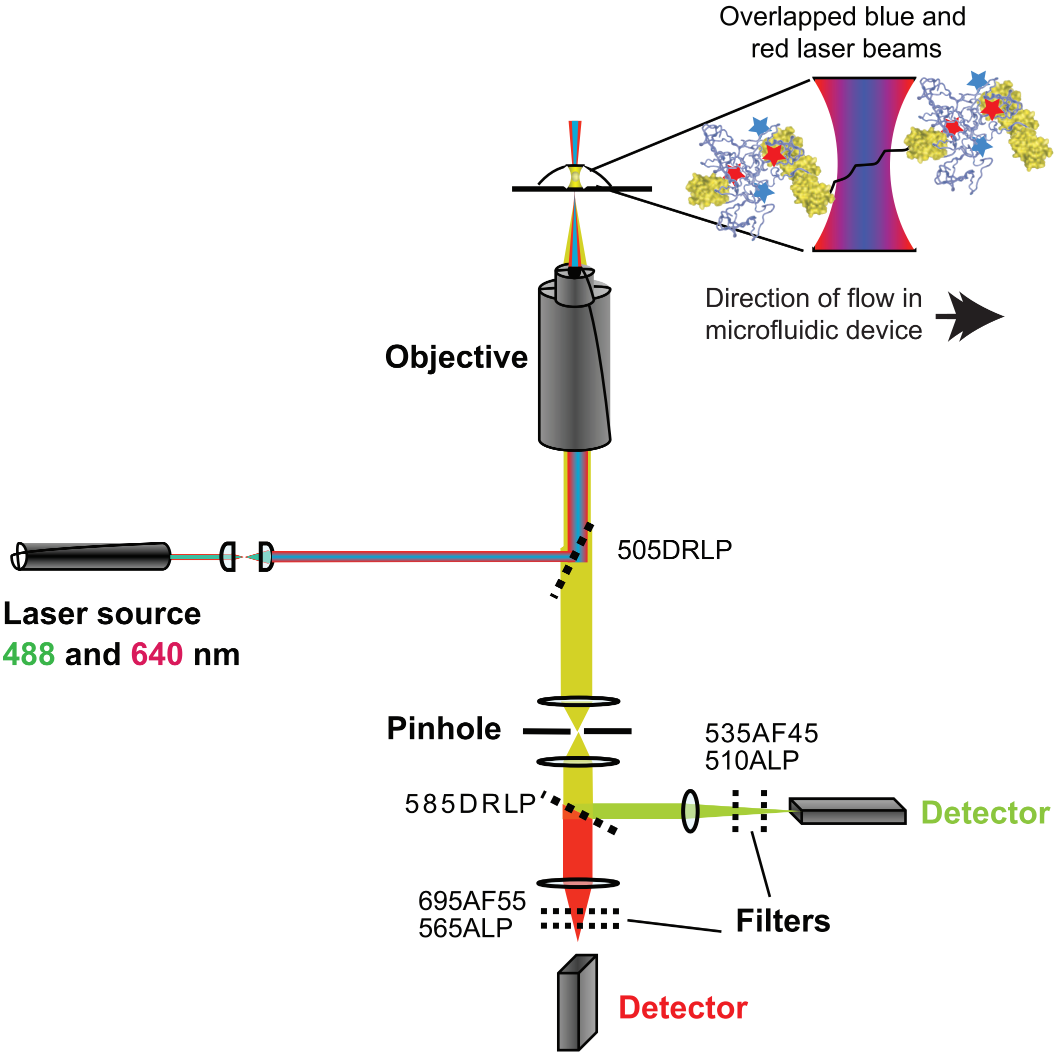

Supplementary Figure 3

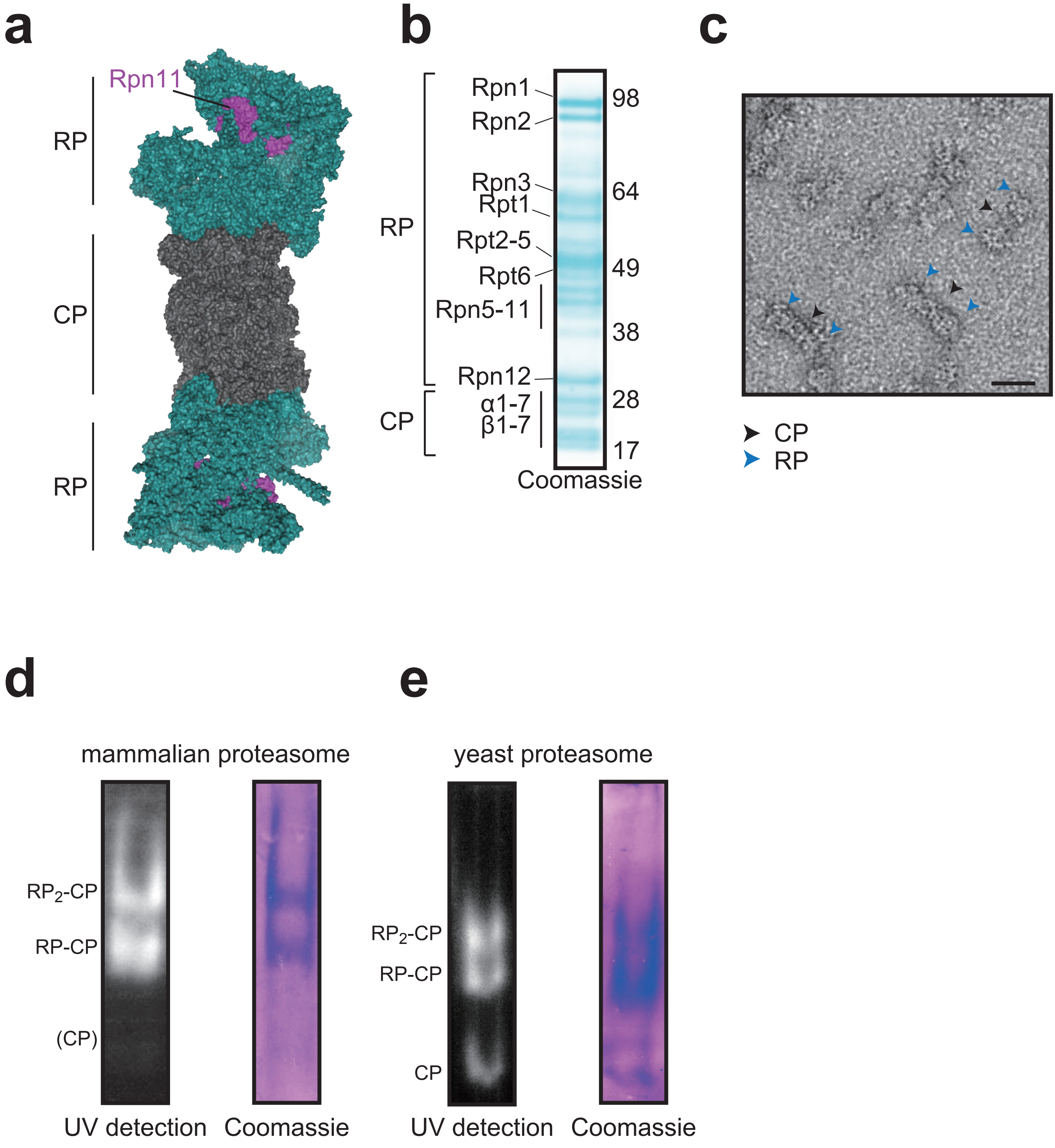

Supplementary Figure 4

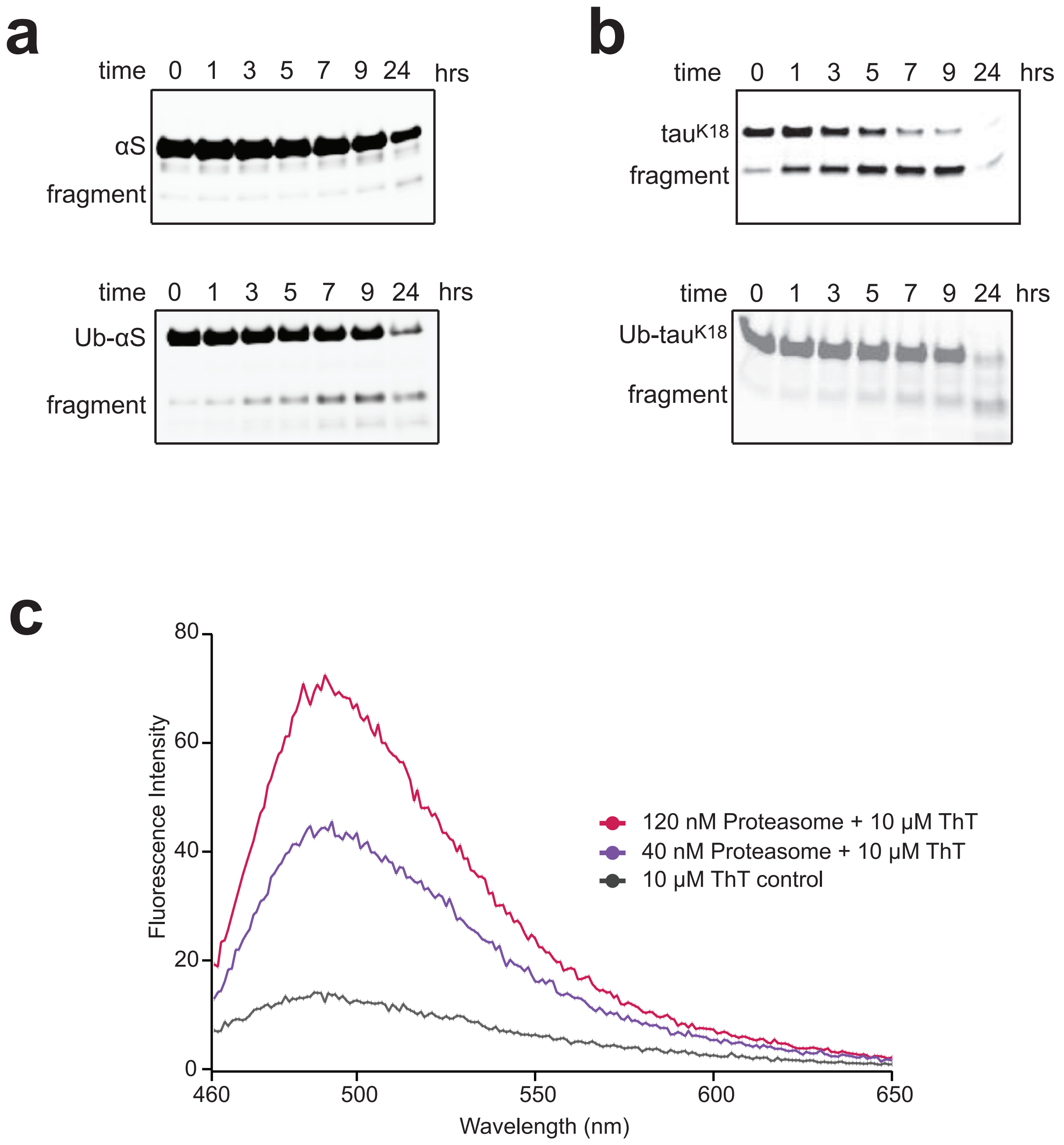

Supplementary Figure 5

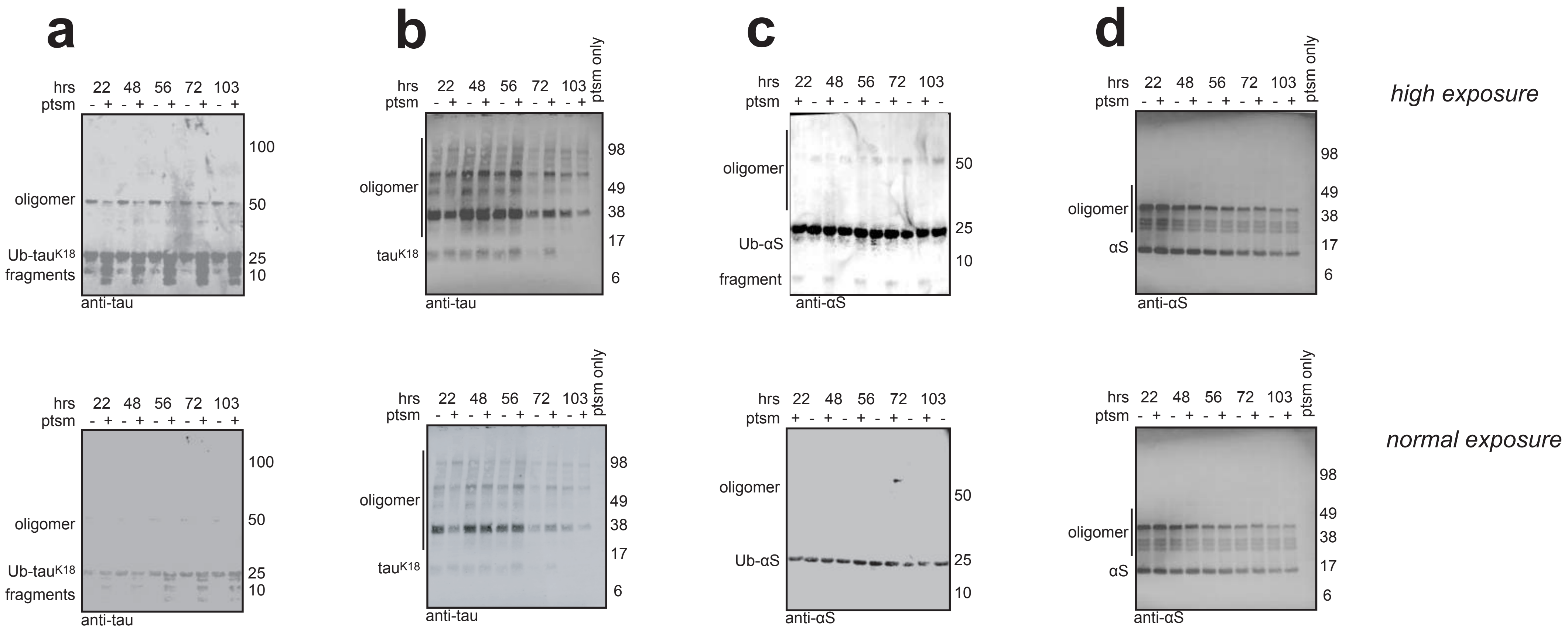

Supplementary Table 1

a Size dependency of Ub-tau^{K18} aggregates removed by the proteasome

Aggregates	4 hrs aggregation Control	4 hrs aggregation +Proteasome	4 hrs aggregation degraded	24 hrs aggregation Control	24 hrs aggregation +Proteasome	24 hrs aggregation degraded	48 hrs aggregation Control	48 hrs aggregation +Proteasome	48 hrs aggregation degraded	72 hrs aggregation Control	72 hrs aggregation +Proteasome	72 hrs aggregation degraded	96 hrs aggregation Control	96 hrs aggregation +Proteasome	96 hrs aggregation degraded
**Small**	1023	57	**94%**	9284	519	**94%**	5234	292	**94%**	2624	1542	**41%**	272	94	**65%**
**Medium**	24	0	**100%**	456	36	**92%**	374	16	**96%**	224	116	**48%**	4	4	**0%**
**Large**	6	0	**100%**	62	6	**90%**	62	10	**84%**	32	18	**44%**	2	0	**100%**

b Size dependency of Ub-αS aggregates removed by the proteasome

Aggregates	4 hrs aggregation Control	4 hrs aggregation +Proteasome	4 hrs aggregation degraded	24 hrs aggregation Control	24 hrs aggregation +Proteasome	24 hrs aggregation degraded	48 hrs aggregation Control	48 hrs aggregation +Proteasome	48 hrs aggregation degraded	72 hrs aggregation Control	72 hrs aggregation +Proteasome	72 hrs aggregation degraded	96 hrs aggregation Control	96 hrs aggregation +Proteasome	96 hrs aggregation degraded
**Small**	6230	1512	**76%**	2898	652	**78%**	2120	812	**62%**	1677	912	**46%**	3474	1765	**49%**
**Medium**	242	70	**71%**	30	8	**73%**	38	24	**37%**	54	34	**37%**	238	86	**64%**
**Large**	8	0	**100%**	2	0	**100%**	0	4	**N/A**	6	0	**100%**	14	24	**N/A**
